# Precision Mental Health Care for Cannabis Use Disorder: Utility of a bioSocial Cognitive Theory to Inform Treatment

**DOI:** 10.3389/fpsyt.2021.643107

**Published:** 2021-06-28

**Authors:** Matthew J. Gullo, Zoë E. Papinczak, Gerald F. X. Feeney, Ross McD. Young, Jason P. Connor

**Affiliations:** ^1^National Centre for Youth Substance Use Research, The University of Queensland, Brisbane, QLD, Australia; ^2^Alcohol and Drug Assessment Unit, Princess Alexandra Hospital, Brisbane, QLD, Australia; ^3^School of Psychology, The University of Queensland, Brisbane, QLD, Australia; ^4^Jamieson Trauma Institute, Metro North Health, Herston, QLD, Australia; ^5^Discipline of Psychiatry, Faculty of Medicine, The University of Queensland, Brisbane, QLD, Australia

**Keywords:** reward, impulsivity, expectancies, self-efficacy, cannabis, social cognition, *bioSocial Cognitive Theory*, precision medicine

## Abstract

Globally, cannabis is the most frequently used controlled substance after alcohol and tobacco. Rates of cannabis use are steadily increasing in many countries and there is emerging evidence that there is likely to be greater risk due to increased concentrations of delta-9-tetrahydrocannabinol (THC). Cannabis use and Cannabis Use Disorder (CUD) has been linked to a wide range of adverse health outcomes. Several biological, psychological, and social risk factors are potential targets for effective evidence-based treatments for CUD. There are no effective medications for CUD and psychological interventions are the main form of treatment. Psychological treatments based on Social Cognitive Theory (SCT) emphasize the importance of targeting 2 keys psychological mechanisms: drug outcome expectancies and low drug refusal self-efficacy. This mini-review summarizes the evidence on the role of these mechanisms in the initiation, maintenance, and cessation of cannabis use. It also reviews recent evidence showing how these psychological mechanisms are affected by social and biologically-based risk factors. A new *bioSocial Cognitive Theory (bSCT)* is outlined that integrates these findings and implications for psychological cannabis interventions are discussed. Preliminary evidence supports the application of bSCT to improve intervention outcomes through better targeted treatment.

## Introduction

Cannabis is the most widely used illicit drug worldwide and more jurisdictions are decriminalizing or legalizing use (1, 2). In 2018, it was estimated that 3.9% of the global adult population reported past-year cannabis use [~192 million people (3)]. North America (12.4%), West and Central Africa (12.4%), and Oceania (10.3%) are among the highest cannabis-using regions in the world, with rates of use increasing in many nations (3). Cannabis may be more harmful now than it has ever been, due to increased *delta-9-tetrahydrocannabinol* (THC) content—the key psychoactive ingredient (3–5). Higher THC potency cannabis is associated with an increased incidence of adverse side effects (6, 7).

The most severe and likely adverse side effect of cannabis use is developing a Cannabis Use Disorder [CUD; (8)]. Of those who have ever tried cannabis, 1 in 10 will develop moderate-severe CUD, formerly labeled *cannabis dependence* in the DSM-IV (9, 10). This risk increases to 1 in 6 if use commenced during adolescence (11). Daily users hold the greatest risk with ~1 in 2 developing moderate-severe CUD (12). Moderate-severe CUD impacts several areas of functioning with those affected more likely to experience comorbid psychiatric problems, relationship and financial difficulties, insomnia, withdrawal symptoms, reduced energy, low self-esteem and self-confidence, and reduced productivity (13–15). There are no effective medications for CUD and psychological interventions are the main form of treatment (2, 16).

The effectiveness of *cognitive-behavior therapy* (CBT), *motivational enhancement therapy* and *contingency management* as treatment for CUD is well-established [e.g., (16–18)]. CBT for substance use disorder and, to a lesser extent, motivational enhancement therapy with its focus on self-efficacy, are based on Social Cognitive Theory (SCT) (19). Social Cognitive Theory (SCT) conceptualizes cannabis use as a learned behavior that is believed to serve some adaptive and coping functions (e.g., stress reduction, social facilitation). CBT targets the (perceived) functional role that cannabis use plays in a patient's life and seeks to alter the cognitive and behavioral mechanisms precipitating use (20, 21). Patients are taught skills to aid cannabis reduction/cessation and maintain this change. This could involve, for example, teaching patients to identify situations likely to trigger motivation to cannabis use and how to avoid them, or how to address the thoughts and emotions underlying the motivation to use (22, 23). Other components of CBT include building drug refusal skills and problem-solving skills, and making healthy lifestyle modifications (24). The main goals of CBT are to increase patient self-efficacy to resist cannabis use and expand their repertoire of coping skills (21). While effective, CBT and other evidence-based treatments produce modest long-term outcomes in moderate-severe CUD (25–27), less than one third of those with CUD seek treatment and, among those, almost half prematurely discontinue treatment (26, 28–31). Further refinement of effective treatments like CBT could lead to improved patient retention and outcomes. The aim of this mini-review is to summarize the evidence on key psychological mechanisms in CUD and how they are affected by social and biologically-based risk factors. A theoretical review was conducted on published studies of Social Cognitive Theory and cannabis use, encompassing related relevant literature on other drug expectancies, self-efficacy, and temperament/personality. There were no a priori restrictions on the type of published studies included. In integrating these findings, a new *bioSocial Cognitive Theory (bSCT)* is reviewed that could facilitate a more precise application of evidence-based treatments like CBT.

## Social Cognitive Theory

In its application to substance use, SCT predicts that the likelihood of using substances is the result of an individual's drug outcome expectancies and refusal self-efficacy beliefs (32–34). These beliefs can develop through vicarious conditioning (observing others), even before substance use is initiated (19). Individuals who have never used cannabis already hold beliefs about the expected positive and negative outcomes of use, which are called cannabis outcome expectancies, and these beliefs predict future use (35–37).

### Cannabis Outcome Expectancies

Cannabis outcome expectancies are beliefs that an individual holds regarding the expected consequences of engaging in cannabis use, which may be positive or negative (19, 37). Positive cannabis expectancies play an influential role in motivating substance use, whilst negative expectancies generally serve to inhibit use (35, 37–40). Their effect on cannabis use behavior may not be equal. Some studies have found negative cannabis expectancies are no longer associated with consumption when controlling for the effects of positive expectancies (37, 39, 41). Negative expectancies are also a stronger correlate of cannabis-related problems in clinical samples. Therefore, high negative expectancies may be more the result of problematic cannabis use rather than low negative expectancies being an initial cause (37, 41–43).

Expectancies affect motivation to attempt, and ability to succeed in, cannabis cessation. Positive cannabis expectancies are associated with less positive *cessation* expectancies (i.e., beliefs that quitting cannabis will result in positive outcomes), while negative expectancies are associated with more positive cessation expectancies and perceived benefit of reducing use (44, 45). Boden et al. (39) found baseline positive cannabis expectancies predicted greater odds of lapse/relapse during a self-initiated cessation attempt in military veterans with CUD. Negative expectancies predicted lower odds of lapse/relapse. In moderate-severe CUD outpatients, Gullo et al. (46) found that higher levels of negative expectancies predicted greater likelihood of abstinence and fewer days of use over 6 weeks of CBT. While positive expectancies did not directly influence cannabis use, their effect was fully mediated by a negative association with cannabis refusal self-efficacy. That is, positive expectancies may increase relapse risk by undermining confidence in the ability to resist cannabis in cued situations.

### Cannabis Refusal Self-Efficacy

Cannabis refusal self-efficacy is the confidence that an individual has in their ability to resist or refuse using cannabis in cued situations (47). Generally speaking, the strength of self-efficacy beliefs determine whether a person will attempt to cope with a difficult situation and how much effort is exerted (19, 48). Cannabis refusal self-efficacy plays an important protective role at several stages of cannabis use. For instance, high levels of cannabis refusal self-efficacy are associated with non-use in adolescents (49–51). Among frequent cannabis users, high levels of refusal self-efficacy are associated with fewer cannabis-related problems, less severe dependence and fewer days of use (41, 42, 47, 52).

Cannabis refusal self-efficacy may play a role in motivating behavior change among heavy cannabis users. One study found that cannabis refusal self-efficacy was associated with greater readiness to change, and predicted initiation of behavior change among men with cannabis dependence (53). Another study revealed that in female users who had previously tried to quit cannabis, refusal self-efficacy was associated with greater motivation to try again (54). These findings are consistent with Bandura's (19, 48) conceptualization of self-efficacy whereby individuals are more likely to engage in behaviors in which they are confident that they can enact successfully.

Cannabis refusal self-efficacy is consistently associated with better treatment response. Pre-treatment levels of cannabis refusal self-efficacy are associated with greater odds of abstinence during CUD treatment (46, 53) and predict less cannabis use and fewer cannabis-related problems for up to 6 months post-treatment (55). Post-treatment levels of cannabis refusal self-efficacy have an even stronger positive effect, predicting less cannabis consumption at 3, 6, and 12-months post-treatment (56–58). Low self-efficacy in response to negative emotion may be particularly salient (46).

Cannabis refusal self-efficacy may also be an important mechanism of change in treatments for CUD. Several studies have demonstrated that changes in cannabis refusal self-efficacy that occur during treatment are the strongest predictor of long-term abstinence—up to 14 months (25, 56–58). Regardless of specific treatment received, individuals who report the greatest improvements in cannabis refusal self-efficacy in treatment experience the most successful outcomes (33, 58, 59). These results indicate that increased cannabis refusal self-efficacy is a mechanism of change in psychological treatments for CUD.

One study has examined the means through which cannabis refusal self-efficacy may translate into improved treatment outcomes. Litt and Kadden (58) combined data from 3 cannabis treatment trials (*N* = 901) and found that the effects of refusal self-efficacy on cannabis use and cannabis-related problems were partially mediated by increased use of coping skills and by reductions in emotional distress. These findings support Bandura's (19, 48) hypothesis that self-efficacy determines whether a person will attempt to cope with a difficult situation and how much effort is exerted. However, the indirect/mediated effects reported by Litt and Kadden were small and the larger direct effects of self-efficacy remained unexplained. Further research is needed to obtain a better understanding of precisely how increased self-efficacy leads to better treatment outcomes.

Consistent with Bandura's (19, 48) contention that self-efficacy is the final pathway that influences human behavior, cannabis refusal self-efficacy has been found to mediate the effects of other psychological risk factors on cannabis use and related problems: cannabis outcome expectancies, cannabis coping motives and descriptive peer norms (41, 46, 52, 60). Despite the importance of cannabis expectancies and refusal self-efficacy, there is a paucity of research examining these constructs together. This is also true of the wider substance use literature. Theoretically, outcome expectancies should affect refusal self-efficacy in the development of CUD (33). For example, an individual expecting greater reinforcement from cannabis (high positive expectancies) is more likely to believe it to be harder to resist in cued situations (low refusal self-efficacy). The impact of cannabis expectancies on consumption is likely to be mediated in large part by self-efficacy, and this has been demonstrated empirically (41, 46).

In summary, SCT provides a valuable framework to conceptualize CUD and already informs evidence-based treatments. Empirical studies of SCT applied to cannabis use show that the aggregate positive outcomes an individual expects from cannabis, the more likely they are to engage in problematic use. Conversely, the stronger the negative outcomes expected and the more confident that an individual is in their ability to resist using cannabis, the more likely they are to abstain. Positive cannabis expectancies may have a stronger impact on behavior than negative expectancies. However, the important role of refusal self-efficacy as a mediator of expectancy effects complicates simple interpretations, and there is a need for more integrative research to advance the field. Increasing self-efficacy is a primary goal of existing evidence-based treatments. A better understanding of the factors that strengthen refusal self-efficacy could serve to improve upon them.

## bioSOCIAL Cognitive Theory of Temperament, Outcome Expectancies, and Refusal Self-Efficacy

**Figure 1:**
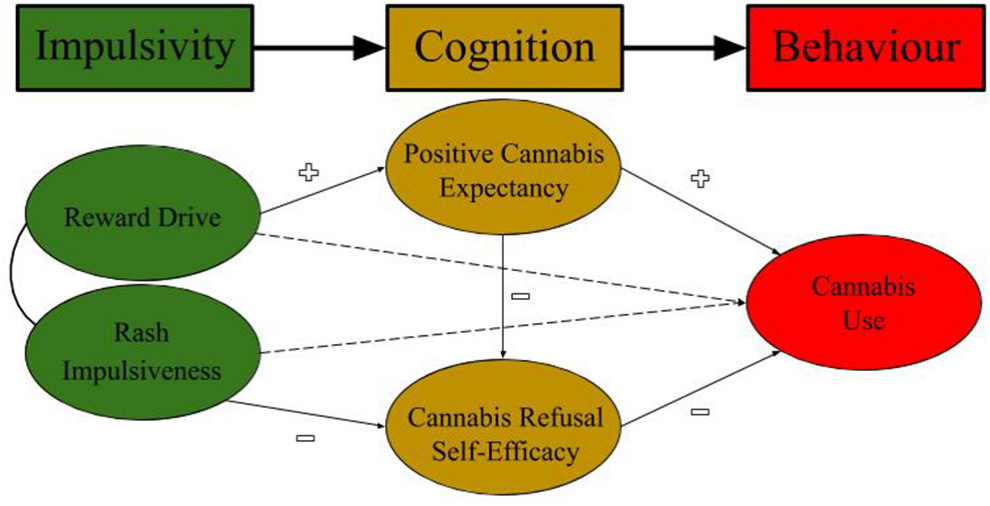
bioSocial Cognitive Theory (bSCT) of Cannabis Use.

Individual differences exist in the strength of one's drug outcome expectancy and refusal self-efficacy beliefs. According to Bandura's (19, 48) notion of triadic reciprocal causation, these beliefs are influenced by, and in turn influence, one's *behavior*, their *environment* and *personal factors* within the individual. Gullo et al. (61) proposed that, when applied to substance use, biologically-based personality traits, specifically reward sensitivity/drive and rash impulsiveness, should act as important personal factors affecting social cognition and behavior (61). These traits are robust predictors of cannabis use (62, 63) and studies have found selective associations between reward drive and positive cannabis expectancies on the 1 hand, and rash impulsiveness and cannabis refusal self-efficacy on the other (51, 64). This *bio*Social Cognitive Theory (bSCT) of temperamental risk factors, outcome expectancies, and drug refusal self-efficacy is depicted in Figure 1.

Reward drive reflects individual differences in one's sensitivity to reward stimuli and subsequent motivation to approach and obtain them, including substances (65–67). Individual differences in reward drive are biologically-based, reflecting variation in mesolimbic dopamine system functioning (68–70). Higher reward drive predicts greater reactivity to substance-related cues and unconditioned responses to their ingestion (71–75). As a result, individuals high in reward drive are more likely to attend to, encode, and recall reinforcement from cannabis use, creating stronger positive outcome expectancies. Studies of young adults and cannabis users referred to treatment have indeed observed a selective association between individual differences in reward drive and positive cannabis expectancies (51, 64). In moderate-severe CUD, positive expectancies are related to poorer treatment response because of their association with lower refusal self-efficacy (46).

Rash impulsiveness reflects individual differences in the capacity to inhibit/modify prepotent approach behavior in light of potential negative consequences (65, 67). Individual differences in rash impulsiveness are biologically-based, reflecting variation in the functioning of the orbitofrontal and anterior cingulate cortices (70, 76–78). Higher rash impulsiveness is associated with poorer reversal learning (79, 80), inhibitory control deficits in substance-dependent individuals (81), and mediates behavioral disinhibition associated with a family history of alcohol use disorder (82, 83). Individuals high in rash impulsiveness are typically aware of their difficulties with inhibitory control, which increases the likelihood of developing a generalized lower self-efficacy for situations requiring reward refusal, including substances (80). This lowered self-efficacy, in turn, increases the likelihood of cannabis use, further exacerbating risk (48). Studies of young adults and cannabis users referred to treatment have indeed observed a selective association between individual differences in rash impulsiveness and lower refusal self-efficacy (51, 64). In moderate-severe CUD, lower cannabis refusal self-efficacy predicts poorer response to treatment (46, 58).

## Discussion

### Toward Precision Mental Health Care for Cannabis Use Disorder

The etiology of CUD is complex and several risk factors have been identified. bioSocial Cognitive Theory (bSCT) synthesizes some of the key risk factors in a way that may help practitioners better understand their combined and interacting effects, including how they manifest in the patient in front of them. This understanding is essential to optimizing treatment (i.e., precision mental health care (84, 85)]. In this mini-review, our focus started in the clinic with an established treatment (CBT) and its proposed mechanism of action (social cognition). We then broadened this focus to incorporate biologically-based factors theorized to directly affect these modifiable mechanisms (impulsivity traits). In outlining the interactions between these biological and cognitive factors, and their effect on behavior, bSCT can reveal individualized targets for CUD treatment.

bioSocial Cognitive Theory (bSCT) proposes modifiable pathways of risk that may be altered directly or indirectly during CUD treatment for different patients. For example, while it is known that increasing refusal self-efficacy is important, the most effective means of doing so will vary between patients (33, 58, 86). Directly increasing it with refusal skills training will work for patients who need to learn skills in how to assertively say “no” when offered or pressured to use cannabis (87, 88). But, according to bSCT, it is less likely to be effective in isolation for those high in rash impulsiveness and holding strong positive expectancies (51, 64). Such patients would be likely to face more significant challenges saying “no” in the first place, because of the greater salience of expected short-term reinforcement (e.g., intoxication) and lesser salience of future negative consequences (e.g., negative urine drug test result at work). For these patients, cognitive restructuring to reduce positive expectancies and strategies to increase reflection and problem-solving would be indicated, according to bSCT. An assessment of patient bSCT factors could reveal high-value therapeutic targets, facilitating tailored treatment.

As a proof-of-concept for bSCT's utility in precision mental health care, Papinczak et al. (89) drew on bSCT to develop a theoretically-driven instant assessment and feedback system (iAx) for CUD. iAx electronically administers and instantly scores validated, standardized assessments and synthesizes this information through the theoretical lens of bSCT. Compared to treatment-as-usual, which administered the same assessments, iAx-enhanced brief intervention led to significantly greater motivation to reduce cannabis use in 87 non-treatment seeking users referred for assessment. Papinczak et al. proposed that iAx may have improved practitioners' formulation of the case, increasing treatment precision. Amidst a sea of assessment results, iAx may have provided a clearer focus on the modifiable factors likely to maximize outcomes for that patient. A CUD and alcohol use disorder version of iAx is now freely available at gullo.com.au/iaxsite.

This mini-review summarizes evidence on the application of bioSocial Cognitive Theory (bSCT) to cannabis use disorder (CUD). Findings are encouraging and consistent with those previously reported in alcohol use disorder (61, 90–92). Further examination of temporal dynamics and reciprocal causation would strengthen clinical application, as there is some evidence of differences in bSCT pathway strength across the continuum of addiction (51, 64). The role of craving is also yet to be explicitly outlined, despite its importance to CUD. Recent developments in cognitive theories of craving and its measurement will facilitate integration (93, 94). There is also scope for inclusion of more fundamental biological factors, such as genetics, building on earlier work in SCT (95, 96), identification of genetic associations with impulsivity and psychiatric comorbidity (97, 98), and incorporating recent methodological advances [e.g., polygenic risk scores (99)]. However, the utility of bSCT in its current form is clear. It provides a coherent theoretical framework for integrating SCT and impulsivity theories of addiction, pointing toward new avenues for targeted treatment. bSCT constructs are predictive of CUD risk, motivation to seek treatment, and response to treatment. Preliminary evidence shows that simply presenting clinical assessment data through the lens of bSCT enhances delivery of brief intervention. These are valuable initial steps toward developing greater precision in the treatment of CUD.

## Author Contributions

MG and ZP created the outline of the review and wrote the first draft. ZP conducted the literature review. All authors contributed to subsequent revisions and approved the final manuscript.

## Conflict of Interest

The authors declare that the research was conducted in the absence of any commercial or financial relationships that could be construed as a potential conflict of interest.
